# Distal Proton Shuttle Mechanism of Ribosome Catalysed Peptide Bond Formation—A Theoretical Study

**DOI:** 10.3390/molecules22040571

**Published:** 2017-03-31

**Authors:** Xiaotong Zhang, Yafei Jiang, Qiuyun Mao, Hongwei Tan, Xichen Li, Guangju Chen, Zongchao Jia

**Affiliations:** 1 Key Laboratory of Theoretical and Computational Photochemistry, Ministry of Education, College of Chemistry, Beijing Normal University, Beijing 100875, China; ZhangXiT@hotmail.com (X.Z.); yafei.jiang@mail.bnu.edu.cn (Y.J.); 201631150035@mail.bnu.edu.cn (Q.M.); xcli@bnu.edu.cn (X.L.); 2 Department of Biomedical and Molecular Sciences, Queen’s University, Kingston, ON K7N 3L6, Canada; jia@queensu.ca

**Keywords:** peptide bond formation, Density Functional Theory, proton shuttle mechanism

## Abstract

In this work, we have investigated a novel distal proton shuttle mechanism of ribosome catalyzed peptide bond formation reaction. The reaction was found to follow a two-step mechanism. A distal water molecule located about 6.1 Å away from the attacking amine plays as a proton acceptor and results in a charge-separated intermediate that is stabilized by the N terminus of L27 and the A-site A76 5′-phosphate. The ribose A2451 bridges the proton shuttle pathway, thus plays critical role in the reaction. The calculated 27.64 kcal·mol^−1^ free energy barrier of the distal proton shuttle mechanism is lower than that of eight-membered ring transition state. The distal proton shuttle mechanism studied in this work can provide new insights into the important biological peptide synthesis process.

## 1. Introduction

**Scheme 1:**
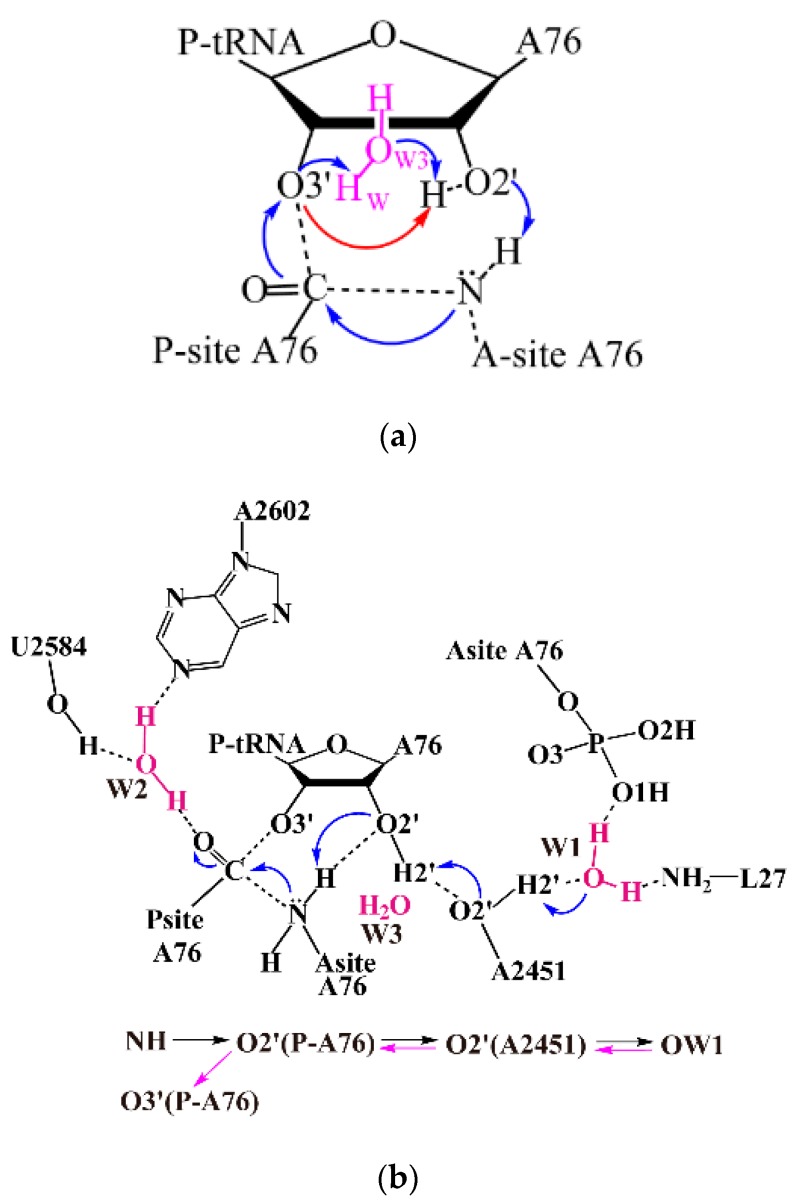
Schematic representation of possible pathways for peptide bond formation. (**a**) concerted six (eight)-membered transition state mechanism; (**b**) distal proton shuttle mechanism. The proton shuttling pattern is shown at the bottom of Scheme 1b. tRNA: transfer RNA.

Ribosomes are responsible for the translation process of the genetic central dogma. The peptide bond formation reaction catalyzed by ribosomes enables essential protein synthesis for all living organisms [1]. As a highly complex cellular machine, ribosome has been extensively studied in both its structure and function. Prokaryotic 70S ribosomes consist of small (30S) and large subunits (50S). The peptidyl transferase center (PTC) of ribosome is located in the 50S. Peptide bond formation reaction proceeds with the aminoacyl-transfer RNA (tRNA, bound to the A site of ribosome) nucleophilic attacking on ester carbon of the peptidyl-tRNA (bound to the P site), accompanied by a proton transfer from α-amino group of aminoacyl-tRNA to deacylated 3′ hydroxyl. A six-membered ring transition state during peptide formation (shown in Scheme 1a) was proposed based on the X-ray structure of the large ribosomal subunit complexed with transition state analogue (TSA), in which the 2′ hydroxyl of A76 in the P site acts as a general base to abstract the proton from the α-amino group and donates another proton to the deacylated 3′ hydroxyl to complete the reaction [2]. Since a water molecule (W3) was found to locate between 2′ and 3′ hydroxyls of A76 in the same crystal structure, an alternative eight-membered proton shuttle transition state (TS-8) was also proposed (Scheme 1a) [2]. Evidence from the kinetic isotope effects (KIE) experiment also indicates that one water molecule becomes involved in the proton transfer process during peptide bond formation [3]. Indeed, proton transport assisted by bridging water is not uncommon and was observed in various enzymatic systems [4,5,6,7,8,9].

In fact, numerous structural water molecules are found in the ribosome structures, several of which reside inside the PTC. It is thus highly plausible that these water molecules play critical roles in peptide bond formation reaction. In addition to W3 located between 2′ and 3′ hydroxyls of A76, a second water molecule (W2) is seen between N of A2602 and 2′ hydroxyl of U2584 in the structure of the *Haloarcula marismortui* (Hma) 50S complexed with substrate analogs [2,10]. Computational mechanism study identified that W2 stabilizes the oxyanion in the transition state via extensive hydrogen bond network centered around this water molecule [11].

Very recently, the structures of pre-attack and post-catalysis complexes of the *Thermus thermophilus* 70S ribosome at ~2.6 Å resolution have been reported. These structures give rise to more details of the ribosomal PTC with bound tRNA substrates and products and ions, especially the water molecules [12]. Both the water molecules mentioned above (W2 and W3) are again observed in these X-ray structures, inferring their critical roles for the ribosomal function. Moreover, a third water molecule, which lies within the cavity formed by residues A2602, A2451 from the 23S rRNA, the 3′ end of the A-site tRNA and the N terminus of protein L27, was observed. This water molecule is not buried within the active center but located about 6.1 Å away from the nucleophilic NH_2_ (W1, Scheme 1b). However, through the hydrogen bond is formed with the 2′-OH of A2451, this water molecule bridges a potent proton transfer pathway for the peptide formation reaction. Compared with the conventional proton transfer pathway that is limited in the active site, this pathway allows the proton on the attacking amine to be shuttled to 3′ hydroxyl of A76 via W1 distally, thus with potentially less steric constrain [12].

Previous studies of the ribosome have emphasized the importance of water, which mediates in proton transfer in peptide formation reaction [11,13,14]. QM/MM (Quantum Mechanics/Molecular Mechanics) simulation performed by Świderek et al. confirmed that a W3-mediated eight-membered ring transition state experiences an energy barrier of 27.60 kcal·mol^−1^, significantly lower than 33.90 kcal·mol^−1^ of the six-membered ring proton shuttle mechanism, in which no water is involved [14]. Kinetic experiments have determined that the energy barrier of ribosome-catalyzed peptide formation is 14.00 kcal·mol^−1^, 13.60 kcal·mol^−1^ lower than that calculated from the eight-membered ring mechanism. Though the difference between calculation and experiment was considered to be associated with the limitations of the DFT (Density Functional Theory) Hamiltonian employed [14], there is another possibility that the reaction may proceed via an alternative proton transfer pathway with lower energy barrier. Furthermore, a noteworthy experimental observation is that the efficient ribosomal peptidyl transfer critically relies on the ribose A2451. However, the role of A2451 has not yet been adequately addressed in previous theoretical investigations [15,16]. Considering that A2451 bridges 2′ hydroxyl of A76 and W1 in the proposed distal proton shuttle pathway, investigations of this new mechanism may shed light on the role of A2451 and its involvement in ribosome function. In view of these facts, by employing DFT M06-2X functional in this work we have studied the mechanism of the peptide formation in ribosome. M06-2X is a high nonlocality functional developed by Truhlar’s group with double the amount of nonlocal exchange. We compared the proposed distal proton shuttle pathway with the conventional eight-membered ring mechanism. The distal proton shuttle pathway turns out to be energetic favorable with an energy barrier of 27.64 kcal·mol^−1^. The distal proton shuttle mechanism also rationalized the experimental result, demonstrating A2451′s important role in peptide formation.

## 2. Results and Discussions

### 2.1. Comparison of Distal Proton Transfer Mechanism and Eight-Membered Ring Mechanism

**Figure 1:**
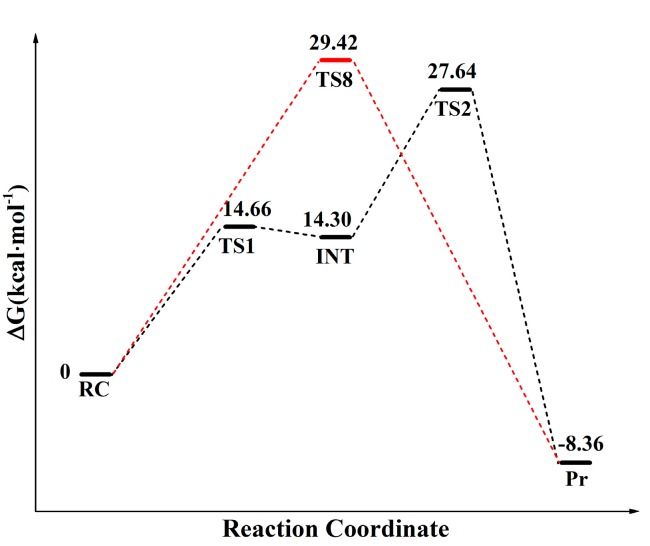
Schematic representation of the M06-2X relative free energy profiles for the peptide bond formation catalyzed by ribosome through distal proton shuttle mechanism and the eight-membered ring proton transfer pathway (TS-8). The relative free energy calculated through the TS-8 and the distal proton transfer pathway is represented in red and black respectively. The solvation effect is estimated by polarizable continuum model (PCM) model with dielectric constant ε = 4. RC: reactant complex; TS1: the first transition state; INT: intermediate; TS2: the second transition state; TS-8: the transition state for eight-membered ring proton transfer pathway; Pr: product.

The calculated free energy profiles of the distal proton shuttle pathway and canonical eight-membered ring pathway are depicted in Figure 1. The energy profile obtained at dielectric constant of 24, 78 are shown in Figure S1. As shown in Figure 1, the free energy barrier of the eight-membered ring transition state is 29.42 kcal·mol^−1^, which is very close to 27.60 kcal·mol^−1^ obtained from QM/MM simulation by Świderek et al. [14], while the distal proton shuttle mechanism follows a two-step mechanism and shows much lower energy barrier of 13.27 kcal·mol^−1^ in the rate-limiting step. The overall free energy barrier of the distal proton shuttle mechanism is 27.64 kcal·mol^−1^, which is also lower than that of the TS-8 pathway. The 11.14 kcal·mol^−1^ overestimation of our calculation with respect to the 16.5 kcal·mol^−1^ experimental values deduced by Sievers et al. may come from the limitations of the DFT Hamiltonian employed to describe the quantum region of the system, which was also observed in previous calculation work [17,18].

**Figure 2:**
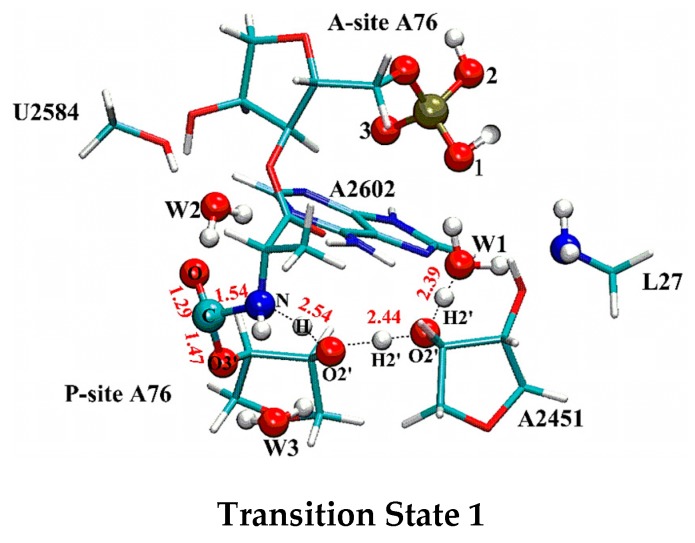
Geometry of TS1 in the distal proton shuttle pathway (Geometries of RC, INT, TS2, Pr are shown in Figure S3). Hydrogen bonds are represented by dashed lines. Important geometric parameters are labeled. The hydrogen bond length is measured as the distance between two heavy atoms (in red).

In contrast to the canonical eight-membered ring mechanism, in which C-N bond formation, C-O (C-O3′ (P-A76) in Figure 2 and Figure S3) bond cleavage and proton transfer are completed in a single step, the distal proton shuttle mechanism uses a two-step strategy. In the first step, the aminoacyl-tRNA nucleophilic attacks on ester carbon of the peptidyl-tRNA with the C-N bond partially formed with the length of 1.54 Å in the first transition state (TS1 shown in Figure 2), accompanied by a concerted proton transferring from the attacking nucleophile to W1 via the P-site A76 and A2451. Since the three protons have been transferred relatively close to their acceptors at the TS1, the proton transferring process has already progressed to the late stage. The calculated Wiberg bond indexes also support this observation. At TS1, the bond indexes between the protons and their approaching acceptors, H-O2′ (P-A76), H2′ (P-A76)-O2′ (A2451) and H2′ (A2451)-OW1 are 0.434, 0.429 and 0.362 respectively, while the bond indexes between these protons and their detaching donors of H-N, H2′ (P-A76)-O2′ (P-A76), H2′ (A2451)-O2′ (A2451) are smaller, taking the value of 0.242, 0.234 and 0.302 respectively. The detailed bond indexes are listed in Table 1. Compared to the reactant complex state, the C-O bond (C-O3’ in Figure 2 and Figure S3) is only slightly weakened at TS1, which elongates 0.13 Å to 1.47 Å. After crossing a 14.67 kcal·mol^−1^ free energy barrier, with the proton being transferred to W1, a negatively charged tetrahedral intermediate (T^−^) centered around C is formed in the PTC. At the intermediate state (INT), the C-O (C-O3′ (P-A76) in Figure S3) bond length of 1.48 Å is almost the same as that in TS1, while the C-N bond further shortens to 1.53 Å. The positive and negative charges become separated in space with the −0.904 negative charge on ester oxygen (O in Figure 2 and Figure S3) and positive charge on the side of the hydronium (0.674). Besides the hydrogen bond with A2451, the hydronium (as shown in Figure S3) is also stabilized by two other hydrogen bonds from 5′-phosphate oxygen of A-site A76 and the N terminus of L27 (with the O_W1_-N_L27_ and O_W1_-O_phos_ distances of 2.56 Å and 2.78 Å respectively, as shown in Table S2). The T^−^ breaks down in the subsequent rate-limiting step by crossing a 27.64 kcal·mol^−1^ free energy barrier. The transition state of this step (TS2) is characterized by the formation of the C-N bond, which continues decreasing to 1.43 Å. At the same time the C-O bond drastically elongates to 1.86 Å. The hydronium releases a proton and shuttles it back to O3′ of P-site A76 through O2′ of A2451 and O2′ of P-site A76. Similar to the first step, the protons are all relayed closer to their acceptors at TS2 (as shown in Figure S3 and bond indexes listed Table 1). In the resulted product, with the proton shuttling back to O3′, the C-N bond is finally formed (with the sustainable increasing bond index of C-N from the RC (reactant complex) to the TS2) and the C-O bond is completely broken.

In the distal proton shuttle pathway, peptide formation reaction follows a two-step mechanism rather than the concerted mechanism found in conventional localized proton transfer pathways through six-membered or eight-membered ring transition states. There is a debate in the literature on whether the ribosome uses a concerted or a stepwise mechanisms. Extensive quantum mechanics calculations have been performed to compare the so-called four-membered, six-membered and eight-membered proton transfer mechanisms of peptide formation [18,19,20]. Though the eight-membered ring TS in which the proton transfer is assisted by a mediate water was identified to be more energetic favorable, they share similar concerted reaction characteristics [13,14]. Without the presence of any intermediate species, C-N bond formation is completed simultaneously with the C-O bond cleavage and proton transfer. Alternatively, several theoretical investigations have also examined stepwise mechanisms of peptide formation, in which C-N bond forms asynchronously with the C-O bond cleavage through a neutral intermediate where the proton from α-amino group is transferred to the carbonyl oxygen atom [11,21,22,23]. There are experimental evidences that the peptide formation reaction proceeds in a stepwise way. A tetrahedral intermediate with S chirality was proposed by Schmeing et al. based on the crystal structures of large ribosomal subunit of Hma complexed with substrate and transition state analogs [2]. By detecting KIE of the reaction, Hiller et al. also found that the peptide bond formation is not fully concerted but proceeds through a stepwise mechanism [24]. However, Kuhlenkoetter et al. performed a kinetic solvent isotope effect (KSIE) experiment to investigate the proton inventory for peptide bond formation, which confirmed formation of three hydrogen bonds with about equal contributions in the rate-limiting step [3]. For all the concerted and stepwise mechanisms studied so far, only the one via eight-membered ring transition state is in accordance with the KSIE experiment. Interestingly, at the TS2 of the distal proton shuttle mechanism, there are also three hydrogen bonds formed among A76, A2451 and W1, which is, to the best of our knowledge, the only stepwise mechanism in agreement with the KSIE results [3].

Another important experimental observation that has not been adequately emphasized in previous mechanism investigations, is the role of the ribose A2451. Erlacher et al. found that the 2′-OH of A2451 is necessary and sufficient to provide almost full catalytic power to the ribosome PTC [15]. In an atomic mutagenesis experiment, Lang et al. also noted that A2451 acts as both hydrogen donor and acceptor in the peptide synthesis. Replacement of its 2′-OH with -F or -OCH_3_ drastically decreases the ribosome’s activity [16]. Though previous mechanism studies confirmed that A2451 can either activate the proton donor function of the 2′-OH of A76 or stabilize the transition state via hydrogen bonding with A76, it is thought to play only a single role as a hydrogen bond donor in the reaction [11,21,22,23]. However, our results show that the 2′-OH of A2451 indeed serves as both the proton donor and acceptor to shuttle the proton between 2′-OH of A76 and W1. The proton departure trajectory of the distal proton shuttle mechanism is clearly in accordance with these experimental observations that A2451 plays crucial role in the peptide formation reaction [15,16].

Though the distal proton shuttle pathway presents very close energy barrier compared to the canonical eight-membered ring mechanism, it may benefit from some aspects energetically. First of all, in the distal proton shuttle pathway, cleavage of N-H bond and C-O bond is completed in separate steps, which avoids the energy barrier accumulation encountered in the concerted eight-membered ring transition state. Furthermore, cleavage of the C-O bond is progressively completed in two steps, as seen from the variation of its Wiberg index and its bond length (Figure 2 and Figure S3 and bond index of C-O3′ (P-A76) in Table 1), which also contributes to the lower energy barriers. The distal proton shuttle also induces less steric tension in its two transition state structures than that of the eight-membered or six-membered ring. As shown in Figure S3, the angle ω between three successive non-hydrogen atoms (N of attacking amine, O2′ of P-tRNA A76, OW3) of eight-membered ring transition state and (N of attacking amine, O2′ of P-tRNA A76, O2′ of A2451) of the TS2 in the distal proton shuttle mechanism are 80.54° and 107.36°, respectively. Clearly, the latter one is much closer to the ideal angle of 109.5° for a tetravalent atom. The electron population of the ribosome is also more feasible for the proton on the α-amino group to remotely transfer to W1 based on the NBO (Natural Bond Orbital) analysis of the key atoms in the reactant complex (Table S1). Compared to the charge of −0.579 on O3′ of P-site A76 (the proton acceptor in eight-membered ring mechanism), the O_W1_ is more negative (−1.035), which makes W1 a better proton acceptor in the reaction. Moreover, the negative charge developed on O2′ (P-A76), O2′ (A2451) and O_W1_ along the proton transfer pathway are −0.828, −0.841, −1.035 respectively, facilitating proton transfer along the hydrogen bond chain. By contrast, at the intermediate state, the charges on O2′ (A2451), O2′ (P-A76), and the final proton acceptor O3′ (P-A76) decrease to −0.855, −0.866 and −0.678 respectively, promoting proton transfer back to O3′ (P-A76) in the rate-limiting step.

### 2.2. The Important Role of Water Molecules in the Peptide Formation Reaction

Our calculation indicates that W1 plays a significant role in the distal proton transfer mechanism. The two hydrogen bonds formed with 5′-phosphate oxygen of A-site A76 and the N terminus of L27 strongly polarize W1, which becomes the proton acceptor in the reaction. At the transition states, the hydronium formed by W1 further interacts with A2602 and A2451 via hydrogen bonding, which undoubtedly stabilizes the transition states and thus lowers the activation energy. In the previous study, W2 has been proven to play a very important role in stabilizing the oxyanion via hydrogen bonding with O2′ of U2584, N7 of A2602 and the ester oxygen of P-site A76 [2]. Actually, W2 is in an even more critical position in the distal proton shuttle mechanism as it provides additional stability to the negative-charged tetrahedral intermediate (T^−^) in the reaction. The hydrogen bond between O_W2_ and O is 2.92 Å in the reactant complex, which decreases to 2.69 Å and 2.67 Å in the TS1 and INT respectively, reflecting its strengthening due to formation of the T^−^. At the TS2, this hydrogen bond elongates back to 2.76 Å along with the proton being shuttled back to O3′ (P-A76). The water W3 has been proposed to play a crucial role in the peptide bond formation by mediating the proton transfer between O2′ and O3′ of P-site A76 in eight-membered ring transition state mechanism. Interestingly, W3 does not seem to be so critical in the distal proton shuttle mechanism. In the TS2 of the rate-limiting step, the O2′ directly transfers its proton to O3′ but bypasses W3. We also located a transition state structure where W3 indeed mediates the proton transferring back from O2′ to O3′ in the second step of the distal proton shuttle pathway (Figure S2), the energy barrier increases by 2.65 kcal·mol^−1^. Though this difference in energy cannot fully eliminate the possibility that W3 is involved in the proton transfer between O2′ and O3′ of P-site A76, it is not consistent with the KISE proton inventory experiment showing that only three hydrogen bonds are formed in the rate-limiting step [3]. Even though W3 does not directly participate in the proton transfer process in the distal proton transfer pathway, it is still in a pivotal position in the PTC. At the RC state, the hydrogen bond from W3 polarizes O2′ so that it is more potent to abstract proton from N (NH_2_). The hydrogen bonds between O_W3_-O2′and O_W3_-O3′ also stabilize the T^−^ formed at TS1 and INT states. From the INT, by strengthening the hydrogen bond between O_W3_ and O3′, W3 again polarizes O3′ to facilitate the proton being shuttled back to the water molecule (Figure 2 and Figure S3).

### 2.3. The Function of L27 and 5′-Phosphate of A-Site A76

The N terminus of L27 and the A-site A76 5′-phosphate are of pivotal importance in the distal proton shuttle pathway. They form hydrogen bonds with W1 and do not only polarize W1 and make it a proton acceptor in the first reaction step, but also stabilize the hydronium in the TS1 and INT states. Though the hydrogen bond between W1 and A2602 is effective against a proton being transferred to W1 in the first step, it helps the hydronium to release a proton back to O3′ in the rate-limiting step (the O_W1_-N_A2602_ distance is shortened by 0.23 Å from INT to TS2, as shown in Table S2). It is also noteworthy that the hydrogen bond from A2602 is partially counteracted by the hydrogen bond between L27 and W1. Since L27 is located on the opposite side of W1, as a result of the strengthened interaction between A2602 and W1, the O_W1_-N_L27_ distance is elongated by 0.18 Å from INT to TS2. It may explain why A2602 was found to have little effect on the peptide formation reaction.

## 3. Materials and Methods

**Scheme 2:**
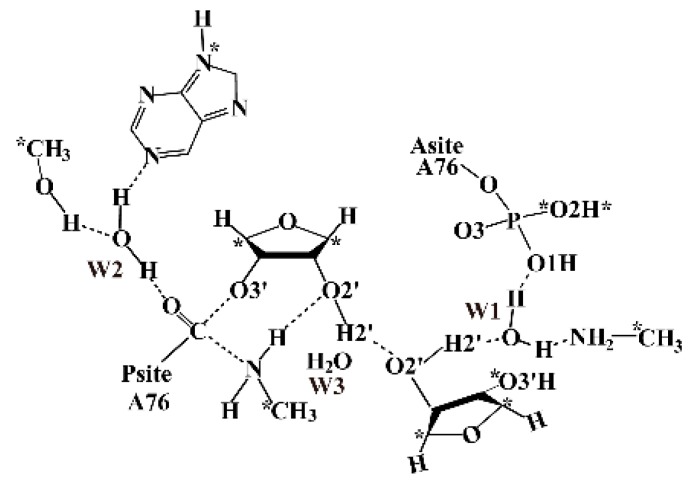
Schematic representation of the Quantum Mechanics model in this work.

The preattack complex structure of the *Thermus thermophilus* 70S ribosome (Protein Data Bank, PDB code: 1VY4) was used to build the QM model for mechanism study [2]. The residues within 50 Å of the PTC were taken from the crystal structure. The energy minimization was performed with AMBER99 force field (2500 step steepest decent followed with 2500 step conjugate gradient optimization) to eliminate the steric conflict introduced by adding hydrogen atoms to the crystal structure. Therefore, all of the heavy atoms of the ribosome were constrained with 50 kcal/mol·Å force during optimization. The optimized structure shows very small root mean squared deviations (RMSD) of 0.32Å with the crystal structure. The QM model was built by extracting all relevant residues in PTC from the optimized structure, which consists of the two substrates (A76 in P site and A site respectively), the water W3, the residues along the distal proton transfer pathway including the ribose A2451, water W1, N terminus of L27 and a phosphate group from A-site A76. Due to that, the water W2 gets involved in the reaction by forming a hydrogen bond with the ester O of P-site A76. It is also included in our model along with the ribose A2602 and U2584, which interact with the W2 with hydrogen bonds. There is an Mg^2+^ (Mg3487) that interacts with the A76 phosphate in the A-site through two water molecules WAT4003 and WAT4004. Due to the positive charge carried by the Mg^2+^, it may profoundly influence the reaction process. Therefore, the phosphate group is protonated on O1 to mimic the electronic static effect of the magnesium ion. The final QM model is shown in Scheme 2, which consists of 120 atoms in total with neutral charge. The atoms marked with asterisks were constrained to their coordinates from the crystal structure.

Geometry optimizations as well as transition state search were then carried out by using the M06-2X functional in combination with the 6-31G (d, p) basis set [25,26]. Based on the located stationary point geometries, high-accuracy energies were further evaluated by the M06-2X functional with larger basis set 6-311 + G (d, p). Zero-point energy (ZPE) effect correction was performed at the same level as the geometry optimizations. Since the dielectric constant of the internal ribosome environment is a subject of debates, the PCM (Polarizable Continuum Model) continuum solvation model with three different dielectric constants (4, 24, and 78) was introduced to estimate for the dielectric effect [27,28]. The final energies reported here are the free energy barriers with the large basis set, which are corrected for ZPE and solvation effects. All QM calculations were carried out with the Gaussian09 program [29].

## 4. Conclusions

In this work, we performed a DFT study on the peptide bond formation reaction catalyzed by the ribosome. By transferring the proton on the attacking amine to a water molecule located about 6.1 Å away, the ribosome utilizes a stepwise strategy to complete the reaction. The calculated energy barrier of the rate-limiting step of the distal proton shuttle pathway is more favorable than the canonical eight-membered ring mechanism and agrees well with that determined from kinetic experiments. In the first step, following the nucleophilic attack on ester carbon of P-tRNA A76, three protons are concertedly transferred from the attacking amine to W1 via O2′of P-site A76 and O2′of A2451. After experiencing a transition state with free energy barrier of 14.67 kcal·mol^−1^, a tetrahedral intermediate and a hydronium are formed at separate positions. In the subsequent rate-determining step, as the result of the peptide bond formation, the hydronium releases the proton back to O3′of P-site A76 via O2′ of A2451, O2′ of P-site A76. The water molecule W1 is of crucial importance in the reaction by acting as the proton acceptor in the intermediate state of the reaction. W2 and W3 are also intimately involved in the reaction through the hydrogen bonds with surrounding residues in PTC. The important role of 2′ hydroxyl group of A2451 and L27 as well as the 5′phosphate of A-tRNA A76 are also examined in the new reaction mechanism.

## Figures and Tables

**Table 1:** Wiberg bond indexes of important bonds in the reaction species (Computed at M06-2X/6-311G+(d, p) level).

Distal Proton Shuttle Mechanism						TS-8 Mechanism
	RC	TS1	INT	TS2	Pr	
N-H	0.775	0.242	0.203	0.035	0.003	0.140
H-O2′(P-A76)	0.027	0.434	0.474	0.451	0.007	0.525
O2′-H2′(P-A76)	0.633	0.234	0.179	0.490	0.646	0.336
H2′(P-A76)-O2′(A2451)	0.055	0.429	0.492	0.151	0.056	0.001
O2′-H2′(A2451)	0.603	0.302	0.207	0.549	0.605	0.619
H2′(A2451)-O_W1_	0.090	0.362	0.460	0.130	0.085	0.081
H-O3′(P-A76)	0.002	0.003	0.003	0.151	0.665	0.001
O_W3_-H_W3_	0.766	0.727	0.725	0.686	0.694	0.407
C-N	0.040	0.815	0.830	0.985	1.215	0.882
C-O3′(P-A76)	1.016	0.767	0.762	0.356	0.001	0.514
C-O	1.694	1.265	1.257	1.473	1.600	1.429

## Supplementary Materials

Supplementary materials are available online.
