# Supplementary material for: Distal Proton Shuttle Mechanism of Ribosome Catalysed Peptide Bond Formation—A Theoretical Study
Source: Molecules. 2017 Mar 31;22(4):571. doi: 10.3390/molecules22040571 (PMC6154465; doi:10.3390/molecules22040571)
Supplement: Supplementary file 1 [file molecules-22-00571-s001.pdf]

Supporting information for " Distal Proton Shuttle Mechanism of Ribosome Catalysed Peptide Bond Formation – A Theoretical study"

Xiaotong Zhang [a], Yafei Jiang [a], Qiuyun Mao [a], Hongwei Tan [*a], Xichen Li [a], Guangju Chen [*a],Zongchao Jia[a, b]
a. College of Chemistry, Beijing Normal University, 100875, Beijing, China.
b. Department of Biomedical and Molecular Sciences, Queen's University, Kingston, Canada K7L 3N6, Canada.

Table S1. NBO charge of the important atoms in the reaction species.

	Distal proton shuttle mechanism					TS-8
	RC	TS1	INT	TS2	P	mechanism
N	-0.951	-0.793	-0.795	-0.778	-0.695	-0.790
H	0.411	0.540	0.541	0.577	0.523	0.557
O2′(P-A76)	-0.828	-0.865	-0.866	-0.787	-0.837	-0.786
H2′(P-A76)	0.547	0.568	0.562	0.585	0.536	0.549
O2′(A2451)	-0.841	-0.848	-0.855	-0.846	-0.840	-0.821
H2′(A2451)	0.539	0.564	0.563	0.553	0.540	0.529
O_{W1}	-1.035	-0.999	-0.985	-1.032	-1.035	-1.039
O3′(P-A76)	-0.579	-0.678	-0.678	-0.811	-0.816	-0.722
O	-0.662	-0.902	-0.904	-0.779	-0.687	-0.813
C	0.901	0.803	0.802	0.781	0.722	0.794
O_{W3}	-1.003	-1.007	-1.008	-1.015	-1.016	-1.025
H_{W3}	0.541	0.500	0.499	0.517	0.508	0.521

Table S2. The key hydrogen bonds in the distal proton shuttle mechanism. (The hydrogen bond length is measured as the distance between heavy atoms.)

	R	TS1	INT	TS2	P
OW1-O(PO_4)	2.94	2.79	2.78	2. 89	2.95
OW1-N(L27)	2.75	2.62	2.56	2.74	2.76
OW1-N(A2602)	2.94	3.16	3.22	2.99	2.93
OW3-O2′(P-A76)	2.92	2.90	2.90	3. 17	2.80
OW3-O3′(P-A76)	2.99	2.84	2.84	2.72	2.78
OW2-N(A2602)	2.84	2.89	2.90	2.82	2.81
OW2-N(U2584)	2.74	2.71	2.71	2.70	2.76
OW2-O	2.92	2.69	2.67	2.76	3.85

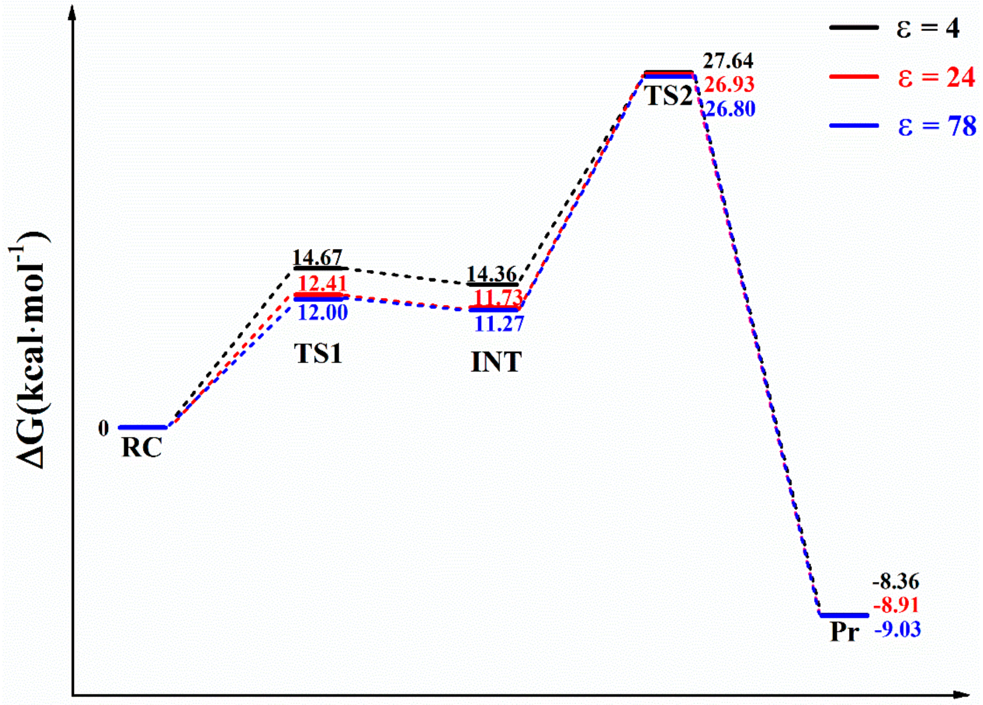

Figure S1. Schematic representation of the energy profiles of the distal proton shuttle mechanism. The PCM continuum solvation model with the dielectric constant of 4, 24 and 78 was introduced to mimic the water environment. ZPE correction is also included. The overall energy barriers are very close under different dielectric constants, which validates the QM model used in this paper.

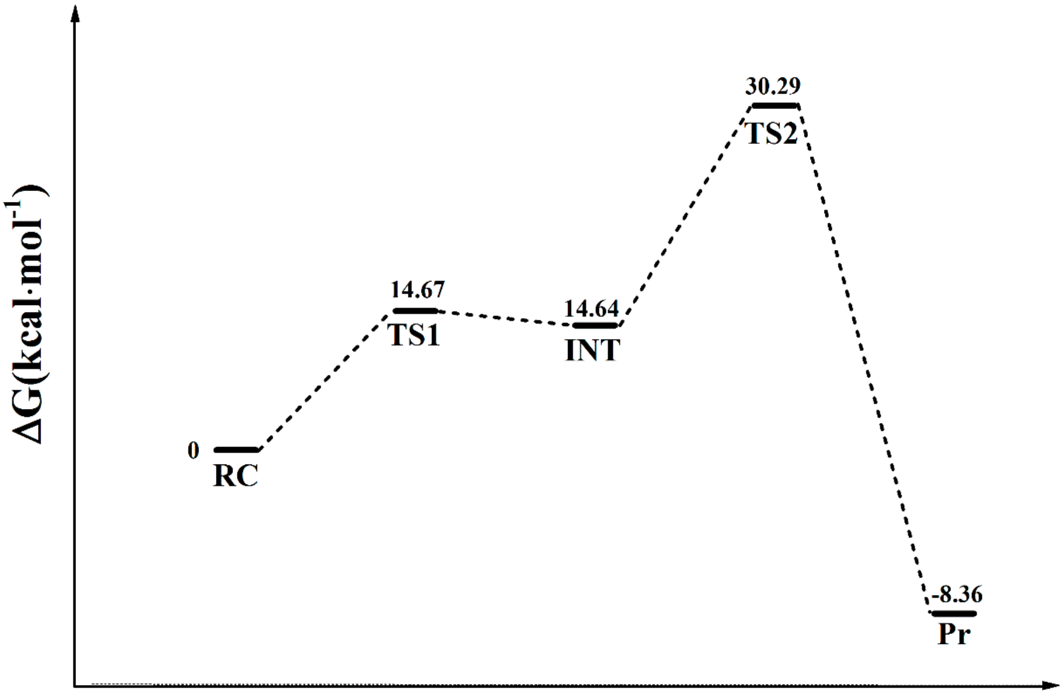

Figure S2. Schematic representation of the energy profile for the peptide bond formation with W3 involved in proton transfer in the rate-limiting step.

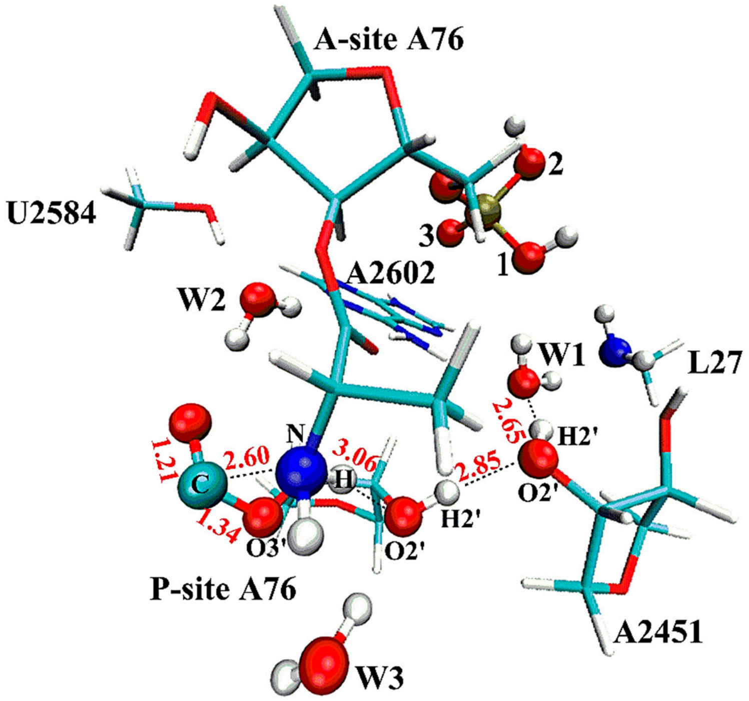
A-site A76
2
3
1
U2584
A2602
W2
W1
L27
2.65
H2'
N
1.21
2.60
3.06
H
2.85
C
O2'
1.34
H2'
O3'
O2'
P-site A76
A2451
W3

RC

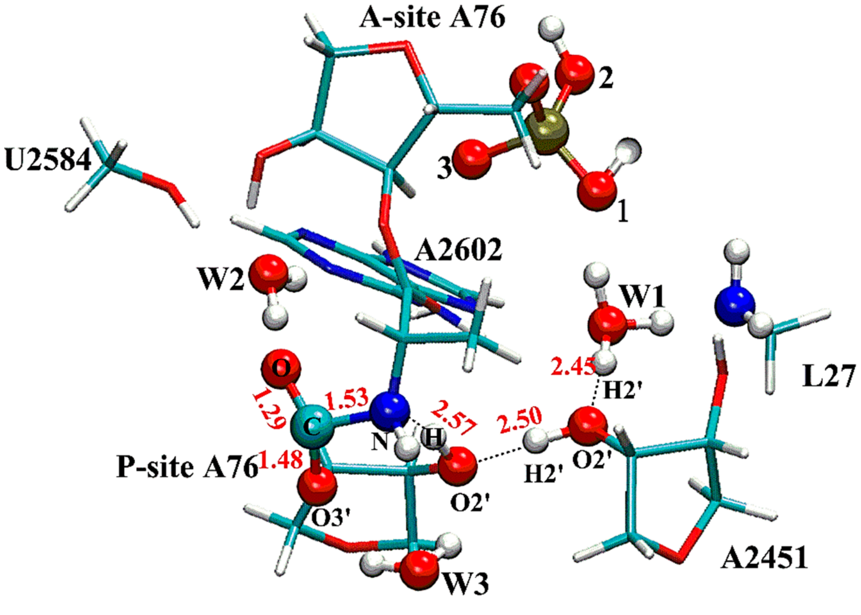
A-site A76
2
3
1
U2584
A2602
W2
W1
L27
O
2.45
H2'
1.29
1.53
2.57
2.50
C
N
H
O2'
P-site A76
1.48
H2'
O2'
O3'
A2451
W3

INT

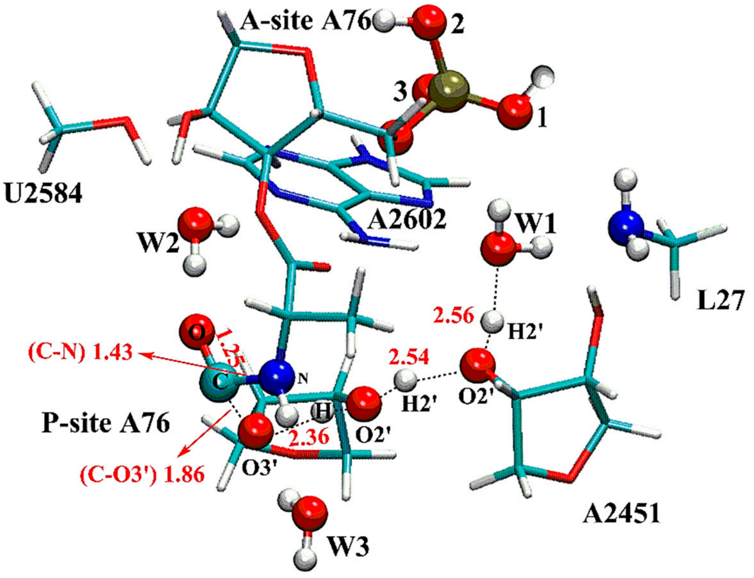

TS2

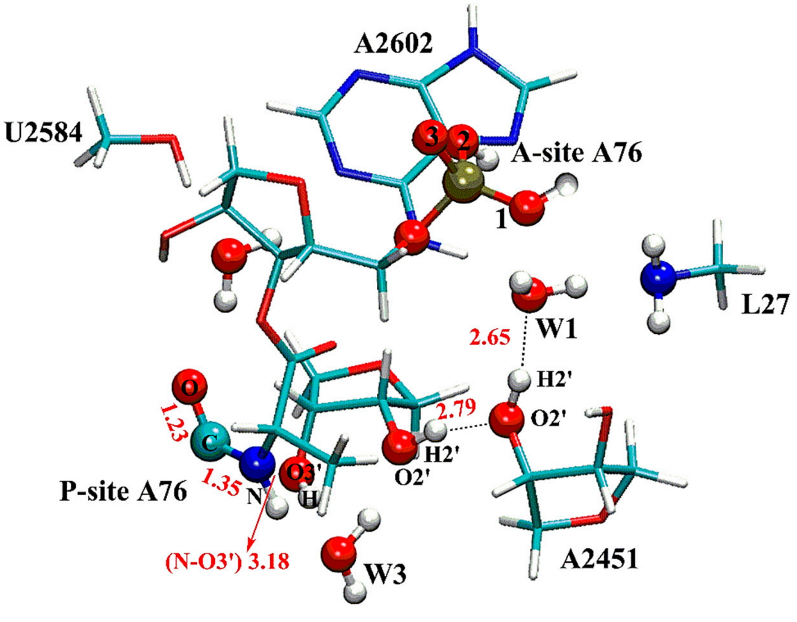

Pr

Figure S3 Geometries of reaction species including RC, INT, TS2 and Pr in the distal proton shuttle pathway. Hydrogen bonds are represented by dashed lines. Important geometric parameters are labeled. The hydrogen bond length is measured as the distance between two heavy atoms (in red).

Table S3. Cartesian coordinates of the RC, TS1, INT, TS2 and P in the distal proton shuttle mechanism.

RC

C	41.77957100	57.43800300	57.79646800
N	41.60336600	57.81613300	56.40908300
H	41.54660900	56.98659100	55.82009000
H	42.45248700	58.29097900	56.10021600
C	42.85814500	56.36465200	57.95264400
H	40.82777800	57.09477100	58.21265500
C	42.61034500	61.98045300	53.98986600
C	42.55775900	60.91368700	55.08594500
C	44.01518600	60.36806400	55.12911800
C	44.65320400	61.07728100	53.92619000
O	43.97206500	62.30553500	53.82357400
O	44.09909900	58.96190100	55.05829200
H	44.79047700	58.64171400	55.67522900
H	44.47306500	60.72701200	56.05512100
O	41.60904300	59.93024400	54.67051300
C	40.44808100	59.88501600	55.34224500
O	40.15156400	60.61463900	56.26118300
C	39.45926100	58.92060100	54.70997200
N	38.54599000	58.45530900	55.73097600
C	38.72858700	57.23202000	56.31632100
O	38.34190100	56.93051300	57.42388000
H	39.23280500	56.51582600	55.63970900
H	38.23501700	59.17417400	56.37854900
C	38.72552600	59.63906800	53.56626300
C	37.78310800	58.69118800	52.83427300
S	36.80241900	59.47733000	51.52381300
C	38.12207100	59.86755800	50.34277200
H	38.69562500	58.97121200	50.09436700
H	37.63909300	60.23421400	49.43613800
H	38.79044400	60.64503900	50.71720500
H	37.05690900	58.28044300	53.54060700
H	38.33858900	57.84701100	52.40832600
H	38.15953700	60.48503600	53.97220500
H	39.47380900	60.04763200	52.87670300
H	40.02523600	58.06953800	54.31840200
H	42.26592300	61.29833600	56.06197800
C	38.75550600	63.69729800	60.33743800
O	40.12462200	63.44595600	60.54694900
H	40.43646200	62.85390700	59.83508000
O	41.25150000	61.76059900	58.69895500

H	42.08167200	62.28329700	58.62388600
H	40.90743200	61.57640600	57.81405600
O	42.24351200	57.28071100	53.56382400
H	41.89003600	57.90158600	52.91855000
H	42.90318600	57.81366700	54.03819700
H	38.55883500	64.18200500	59.37084200
H	38.14812400	62.78251200	60.38964900
H	38.41296100	64.37179000	61.12609900
H	42.19622900	61.53852200	53.07005500
H	42.06171600	62.89275900	54.23005200
H	45.71938900	61.27601200	54.04530500
H	44.49616400	60.45218800	53.03084500
P	46.17745200	61.35835400	61.62943000
O	46.14861900	62.75249800	61.20284100
O	44.72215300	60.72106900	61.82930300
C	44.44282000	59.32548000	61.62600600
H	44.99618500	58.74087200	62.37181100
H	44.75963200	59.03438800	60.61748500
O	46.96153300	60.31573600	60.66109300
O	47.04618300	61.05260200	62.96588200
N	46.56697000	66.25297800	59.07498600
C	47.56243100	65.40206500	58.64635800
H	48.57799000	65.75888000	58.53473500
N	47.18815400	64.17655600	58.38863400
C	45.82965700	64.16099200	58.65562000
C	44.82941200	63.12690500	58.62786300
N	45.04412700	61.83280900	58.35195900
H	44.25396500	61.19799500	58.35879800
H	45.92796400	61.52497200	57.96306500
N	43.55447000	63.39353000	58.92394600
C	43.26162400	64.62633100	59.33385000
H	42.22700000	64.80600100	59.62183200
N	44.10299100	65.61409900	59.43404400
C	45.38976200	65.40521400	59.08779500
N	49.84646500	59.84217100	58.80864700
H	49.56040500	60.17206300	59.72665300
H	49.66522900	58.84236500	58.75810300
C	51.27185900	60.10201000	58.60799800
H	49.02716800	58.60847600	55.59036100
O	48.37065700	57.15035000	54.22937800
C	46.99613200	56.90591800	54.56688800
C	48.39041700	56.74719100	56.50119300
H	48.71059800	55.69801900	56.44132800
C	46.89977600	56.82432700	56.09438800

H	46.35921600	55.92380600	56.40817800
O	46.21522200	57.92506600	56.65264400
H	46.81432900	58.63036300	57.00720700
O	48.65243000	57.28514300	57.78072100
O	47.32334500	60.07313100	57.74995700
H	48.29744800	60.20418800	57.86188500
H	51.44994400	61.17895400	58.62248800
H	51.92405200	59.63434600	59.35635700
H	51.56495500	59.73199000	57.62309200
H	48.18588100	56.73298600	58.41926100
H	46.56685100	61.26230200	63.77908400
H	46.62684900	67.21788900	59.35446300
C	49.09600300	57.52788100	55.38393800
H	50.14200300	57.24924900	55.24760500
H	46.69899500	55.97869400	54.06936800
H	46.35755000	57.72217200	54.20840900
H	43.81009300	56.74620000	57.56700100
H	42.99506300	56.06703700	58.99811700
H	42.57239800	55.47593300	57.38412300
H	47.01478200	60.03860400	58.66820500
C	42.95183100	59.03446100	61.78537700
C	42.02473400	59.69007100	60.74158900
C	40.77560100	60.11873400	61.54639400
C	41.40296000	60.34845600	62.91673800
O	42.50051200	59.44316300	63.05323300
O	39.77695700	59.11344300	61.63375300
H	39.58617300	58.81922800	60.73379500
H	40.36706500	61.05042300	61.14003400
O	41.63008900	58.72368400	59.76213800
C	42.23404700	58.66545100	58.57357000
O	43.10058700	59.42151900	58.18761000
H	42.48059100	60.55312700	60.25338300
H	42.84637400	57.94193000	61.69029600
H	41.76466600	61.38147300	62.99123400
H	40.68623200	60.14869800	63.71557300
H	47.33452200	59.60117700	61.19628900

TS1

C	42.03808000	57.63113200	57.68427400
N	42.25262000	58.08263400	56.31002800
H	42.29369700	57.29450000	55.65886600
H	43.52724100	58.75120200	56.01781900
C	42.85719600	56.36614900	57.95491000
H	40.98828700	57.42074400	57.89352500
C	42.62978700	61.96277100	54.00077800

C	42.63332700	60.93756400	55.16072600
C	44.14339900	60.59435600	55.29084500
C	44.67259100	61.05947800	53.93733100
O	43.97956000	62.25451300	53.69468200
O	44.39578800	59.21527900	55.54693400
H	45.61150400	58.85376700	56.01541400
H	44.58083600	61.21082600	56.08034000
O	41.87176100	59.82438200	54.76643800
C	41.18674800	59.09575800	55.84658600
O	40.75997700	59.78434900	56.84687200
C	40.06574100	58.35589000	55.05440800
N	39.44407100	57.37989800	55.93465500
C	39.85875700	56.08855100	55.99152900
O	39.73373900	55.35104500	56.95098400
H	40.33322800	55.76039300	55.04477200
H	39.08241600	57.76708600	56.80200600
C	39.03403800	59.35616800	54.54046000
C	37.94792900	58.68579400	53.70895100
S	36.61953300	59.80283900	53.16527000
C	37.57369600	60.92254300	52.10484600
H	38.13465800	60.35926300	51.35485000
H	36.85735100	61.56756400	51.59421600
H	38.25561800	61.54719500	52.68413300
H	37.44768000	57.91019900	54.29579500
H	38.37939000	58.19753500	52.82666200
H	38.59893400	59.87378700	55.40174900
H	39.56639600	60.10766000	53.95111800
H	40.51381100	57.80129200	54.21823500
H	42.22682400	61.34018300	56.09196100
C	38.75550200	63.69729900	60.33744100
O	40.12562300	63.39946000	60.46114200
H	40.38163600	62.78912700	59.73997500
O	41.24768300	61.66716700	58.69980400
H	42.06720700	62.17403800	58.56160700
H	41.07093400	61.07602500	57.93597700
O	43.13557300	57.62814200	53.47723900
H	42.51607900	58.36909100	53.40832500
H	43.81078500	58.02886500	54.04353200
H	38.51335100	64.17877200	59.37998500
H	38.12094300	62.80537100	60.43871100
H	38.48840100	64.39084000	61.13912800
H	42.12583500	61.49229800	53.14403500
H	42.12666000	62.89941000	54.24634700
H	45.74545600	61.26396600	53.91641800

H	44.43734900	60.28734800	53.18261800
P	46.16916400	61.36285500	61.63698900
O	46.15331100	62.75437200	61.20335700
O	44.72302600	60.72131800	61.83581600
C	44.44491400	59.31792900	61.65572000
H	44.97827200	58.75423100	62.43162400
H	44.78052300	59.01156000	60.66020500
O	46.96548000	60.32024300	60.66458000
O	47.04522400	61.05415600	62.96814200
N	46.56698400	66.25298100	59.07498600
C	47.56163300	65.40422100	58.64419000
H	48.57631400	65.76301000	58.53029100
N	47.18932300	64.17696300	58.38904400
C	45.83062100	64.16136000	58.65896900
C	44.83113700	63.13087300	58.64087000
N	45.04409000	61.83297200	58.35198100
H	44.26575800	61.18405100	58.37282300
H	45.90365800	61.57074400	57.89298800
N	43.56399200	63.38449700	58.95782000
C	43.26494900	64.61875500	59.35631000
H	42.23109300	64.79360200	59.64849200
N	44.10299600	65.61403000	59.43402700
C	45.38843700	65.40595100	59.08894100
N	49.95415100	59.57644800	59.00792200
H	49.79060800	59.80203500	59.98670100
H	49.95057600	58.56239200	58.91645400
C	51.27194700	60.10201700	58.60801800
H	49.17727600	58.60144800	55.60841500
O	48.32704500	57.29826400	54.21737200
C	46.99616200	56.90576200	54.56678600
C	48.30066100	56.82980900	56.49156300
H	48.44612100	55.74432100	56.40634200
C	46.86670500	57.15418100	56.06512400
H	46.11632600	56.50683800	56.53596800
O	46.59674500	58.51943200	56.34789200
H	47.14340400	59.18086100	57.23497100
O	48.65233500	57.28502000	57.78069200
O	47.58253300	59.98504900	57.96360500
H	48.57779100	59.89013700	58.26033700
H	51.26061600	61.19003300	58.67958500
H	52.08898500	59.71302000	59.22281900
H	51.45739700	59.83304900	57.56706900
H	48.18597800	56.73301100	58.41927200
H	46.56589400	61.26384700	63.78133600

H	46.62469400	67.22213800	59.34019800
C	49.09596700	57.52800900	55.38401300
H	50.09545500	57.10862900	55.24761200
H	46.84568600	55.84401000	54.33653300
H	46.27944100	57.49681700	53.98891600
H	43.91544400	56.55031800	57.74244300
H	42.75073000	56.06066500	58.99912500
H	42.48697100	55.55023100	57.32983900
H	47.06950600	60.13316300	58.77964100
C	42.94970900	59.03525500	61.78864300
C	42.03773700	59.71952900	60.75155600
C	40.77654300	60.12246700	61.54832200
C	41.40200000	60.35000000	62.91900000
O	42.49187800	59.43398500	63.05844500
O	39.77600000	59.11500000	61.63600000
H	39.56198600	58.86019600	60.72935100
H	40.36701600	61.05292700	61.14095800
O	41.68001800	58.81927200	59.69669100
C	42.47681700	58.73525400	58.63059000
O	43.51428500	59.35778000	58.49791900
H	42.49401800	60.61544700	60.32742300
H	42.83821500	57.94399000	61.67946200
H	41.77263100	61.37988200	62.99226900
H	40.68377800	60.15710200	63.71813200
H	47.33355800	59.60273500	61.19854300

INT

C	42.02447700	57.62275800	57.68330900
N	42.22612000	58.07169300	56.30853400
H	42.26559700	57.28608800	55.65532000
H	43.55866900	58.76485600	55.99570000
C	42.85815800	56.36463800	57.95265500
H	40.97885800	57.40217600	57.90712400
C	42.63075800	61.96118500	53.99856200
C	42.63013500	60.93257200	55.15686100
C	44.13945500	60.58342800	55.28208600
C	44.67357500	61.05793900	53.93501100
O	43.98139900	62.25557000	53.69824400
O	44.39733300	59.20747800	55.52380500
H	45.69168600	58.81156300	56.05460100
H	44.57634100	61.19785300	56.07381800
O	41.86057700	59.82371800	54.76836300
C	41.17704800	59.09217300	55.85412500
O	40.75860300	59.78513400	56.85750900
C	40.04179400	58.37151400	55.06359300

N	39.41477600	57.39885300	55.94455200
C	39.82391400	56.10555700	55.99819900
O	39.70389300	55.36834600	56.95876200
H	40.28742300	55.77453600	55.04711900
H	39.07032400	57.78863500	56.81785100
C	39.01805400	59.38543000	54.56130600
C	37.91836700	58.73161500	53.73458000
S	36.59788100	59.86593200	53.20709600
C	37.55740300	60.98500100	52.15083800
H	38.10966200	60.42230000	51.39395900
H	36.84491700	61.64081700	51.64858200
H	38.24808800	61.59832500	52.73182300
H	37.41427800	57.95827200	54.32113100
H	38.33757500	58.24371000	52.84623100
H	38.59518000	59.90412500	55.42803300
H	39.55518900	60.13320200	53.97161000
H	40.47847400	57.81558600	54.22221200
H	42.22975200	61.33798900	56.08998100
C	38.75551000	63.69730100	60.33743700
O	40.12684200	63.40429700	60.45975800
H	40.38201500	62.79298300	59.73874100
O	41.23168400	61.67722600	58.68760600
H	42.05759200	62.17185100	58.55064400
H	41.05949900	61.07326500	57.93105300
O	43.10636900	57.63451400	53.46043500
H	42.49277500	58.38275400	53.42120600
H	43.79030200	58.01360300	54.03121300
H	38.51020200	64.17436100	59.37859100
H	38.12426600	62.80353200	60.44284000
H	38.48721500	64.39279700	61.13710300
H	42.13102900	61.49166100	53.13865100
H	42.12504200	62.89664600	54.24351800
H	45.74669200	61.26204900	53.91882300
H	44.44003300	60.29120100	53.17456300
P	46.18265400	61.37096700	61.62864800
O	46.18230500	62.76350600	61.19759400
O	44.73272400	60.73666900	61.81297600
C	44.45045800	59.33408600	61.62679500
H	44.99163600	58.76562700	62.39397400
H	44.77527400	59.03506200	60.62548200
O	46.98725700	60.32815600	60.66056500
O	47.04618100	61.05260400	62.96589000
N	46.56697500	66.25299900	59.07497700
C	47.56044400	65.40450700	58.64188900

H	48.57508700	65.76329300	58.52744000
N	47.18781800	64.17741000	58.38569500
C	45.82944000	64.16226400	58.65781500
C	44.82954200	63.13412900	58.64092900
N	45.04414000	61.83278200	58.35194700
H	44.24955100	61.20248800	58.33253600
H	45.85866500	61.59977600	57.80292400
N	43.56527400	63.38375400	58.96652900
C	43.26511200	64.61816000	59.36414400
H	42.23209700	64.79238800	59.65953000
N	44.10303100	65.61403400	59.43405200
C	45.38788200	65.40679500	59.08886100
N	49.97890000	59.50627600	59.00231200
H	49.83957200	59.64577500	60.00112700
H	49.99262900	58.50199200	58.82677800
C	51.27181300	60.10203400	58.60801900
H	49.15897900	58.60249600	55.61080100
O	48.33247100	57.28569700	54.21768400
C	46.99611600	56.90588400	54.56687900
C	48.31164800	56.81666400	56.49032600
H	48.47504600	55.73376300	56.41100700
C	46.87587300	57.13563500	56.07130900
H	46.13102800	56.47621600	56.53550500
O	46.62343000	58.49031400	56.39682300
H	47.26566700	59.27147600	57.35289300
O	48.65240100	57.28508600	57.78070400
O	47.68929200	60.02596500	57.98724400
H	48.70454800	59.85969300	58.34170100
H	51.23429800	61.17911400	58.77127100
H	52.10977800	59.68129800	59.16921600
H	51.43121100	59.92252500	57.54421200
H	48.18593000	56.73302400	58.41925300
H	46.56685400	61.26229800	63.77908400
H	46.62528100	67.22251300	59.33893900
C	49.09601300	57.52793300	55.38396100
H	50.10234400	57.12567900	55.24509700
H	46.83408800	55.84987300	54.32005500
H	46.28385800	57.51510100	54.00240500
H	43.91198100	56.55759000	57.72657600
H	42.76517400	56.06352200	58.99946100
H	42.48734900	55.54364300	57.33452400
H	47.13410500	60.22907800	58.76569500
C	42.95659100	59.04935700	61.77320900
C	42.03331400	59.72603500	60.74143500

C	40.77503400	60.12313200	61.54636300
C	41.40295200	60.34845900	62.91676300
O	42.50852500	59.44922200	63.04602300
O	39.77695900	59.11345200	61.63375300
H	39.57121200	58.85110500	60.72721000
H	40.36148000	61.05383100	61.14337900
O	41.67232700	58.81992200	59.69364300
C	42.46263400	58.73580800	58.62047500
O	43.49386100	59.36678400	58.48070700
H	42.48155600	60.62311700	60.31110500
H	42.84810900	57.95741100	61.66638900
H	41.75843000	61.38301500	62.99793500
H	40.68995100	60.13812100	63.71626000
H	47.33451400	59.60118700	61.19628900

TS2

C	41.92392500	57.56827900	57.74262500
N	41.95022400	57.96729400	56.35265400
H	41.98994500	57.18035300	55.71298000
H	43.93438900	58.50590600	54.65431100
C	42.85719600	56.36614900	57.95491000
H	40.91904200	57.29927600	58.09728200
C	42.62978700	61.96277100	54.00077800
C	42.59725700	60.85551500	55.10644100
C	44.11274600	60.51287500	55.23841800
C	44.67259100	61.05947800	53.93733100
O	43.98683400	62.26247500	53.74014200
O	44.39232900	59.12040300	55.38831900
H	45.28346900	58.81237300	55.88580100
H	44.54325600	61.02297300	56.10125700
O	41.80088000	59.77853400	54.70186700
C	40.99891400	58.99368200	55.98323600
O	40.69623100	59.79293800	56.91250600
C	39.81126500	58.44869700	55.14581300
N	39.10845500	57.49117400	55.99362500
C	39.49940600	56.18892900	56.06133500
O	39.34834700	55.45510600	57.01704200
H	39.96486800	55.84493000	55.11516300
H	38.77914200	57.87633900	56.87499000
C	38.87603300	59.56557900	54.69926600
C	37.72772900	59.04532000	53.84408300
S	36.50097000	60.30563900	53.38261100
C	37.54599400	61.40188600	52.38543900
H	38.05260300	60.84008000	51.59649800

H	36.88730900	62.13795000	51.92251500
H	38.28231300	61.92684100	52.99630900
H	37.16498100	58.28378600	54.39137400
H	38.10711700	58.57322900	52.92951600
H	38.49410500	60.07456500	55.59022700
H	39.47673500	60.29395800	54.14865900
H	40.19433000	57.89520300	54.27637400
H	42.20584700	61.22746500	56.05928200
C	38.75550200	63.69729900	60.33744100
O	40.12446600	63.43440700	60.52587700
H	40.42011200	62.83341800	59.81224700
O	41.24775600	61.73825900	58.74335500
H	42.09307100	62.20456500	58.60245300
H	41.05763700	61.13132800	58.00439800
O	43.07908500	57.96083100	53.69193300
H	42.42776100	58.75209700	53.96853200
H	43.32074700	58.05726000	52.76420500
H	38.54645000	64.17226100	59.36874000
H	38.13948800	62.78940500	60.40928900
H	38.43184900	64.38379500	61.12438200
H	42.13929300	61.55100100	53.10596500
H	42.12699800	62.88463200	54.29629800
H	45.74602700	61.26089300	53.95611400
H	44.46319600	60.33360500	53.12837700
P	46.16064100	61.35556200	61.64211300
O	46.13210300	62.74759100	61.20935800
O	44.71444200	60.71231400	61.85030900
C	44.43318100	59.31351200	61.64565800
H	44.97180500	58.73386800	62.40598000
H	44.76054600	59.02302500	60.64217100
O	46.94851800	60.31510400	60.67054300
O	47.04522400	61.05415600	62.96814200
N	46.56698400	66.25298100	59.07498600
C	47.56397800	65.40303800	58.65044600
H	48.57887400	65.76178300	58.53826400
N	47.19254700	64.17544200	58.39827200
C	45.83332300	64.15914100	58.66501300
C	44.83519700	63.12478300	58.64993200
N	45.04409000	61.83297200	58.35198100
H	44.29478000	61.16079100	58.47402600
H	45.96044500	61.52442600	58.05570900
N	43.56568400	63.38361100	58.96073600
C	43.26596500	64.61861000	59.35403100
H	42.23146400	64.79466600	59.64404900

N	44.10299600	65.61403000	59.43402700
C	45.38959200	65.40436700	59.09054900
N	49.92664400	59.68504900	59.01335900
H	49.75404300	59.97499500	59.97220600
H	49.85288200	58.67186200	58.96839200
C	51.27194700	60.10201700	58.60801800
H	49.23632000	58.57804500	55.67688900
O	48.27623900	57.43370200	54.23075900
C	46.99616200	56.90576200	54.56678600
C	48.32859800	56.79195100	56.49347100
H	48.53237700	55.71426400	56.42724700
C	46.87036800	57.02816400	56.07929600
H	46.18725200	56.27861400	56.49983500
O	46.43627800	58.32131300	56.43428900
H	46.99238600	58.89917900	57.08709700
O	48.65233500	57.28502000	57.78069200
O	47.46072000	60.01062800	57.92691500
H	48.43499900	60.03308200	58.15910000
H	51.32892500	61.19165800	58.61698000
H	52.06975700	59.70541900	59.24649600
H	51.45293500	59.76774800	57.58451300
H	48.18597800	56.73301100	58.41927200
H	46.56589400	61.26384700	63.78133600
H	46.62419600	67.22078800	59.34473200
C	49.09596700	57.52800900	55.38401300
H	50.07121400	57.07930000	55.17940600
H	46.92530900	55.85510200	54.25437300
H	46.22279200	57.47922300	54.04712300
H	43.86240200	56.62871500	57.61120500
H	42.89122600	56.09000600	59.01194300
H	42.48116300	55.50735700	57.39274000
H	47.02748200	60.03519300	58.79446700
C	42.93865700	59.03244600	61.78262800
C	42.02543800	59.72333200	60.75065400
C	40.77077000	60.12804500	61.55323900
C	41.40200000	60.35000000	62.91900000
O	42.48245200	59.42320000	63.05566300
O	39.77600000	59.11500000	61.63600000
H	39.60434900	58.82528800	60.73075200
H	40.35687200	61.05922200	61.15191700
O	41.64300700	58.82227200	59.71143000
C	42.41430900	58.71208900	58.62270900
O	43.44099500	59.33678200	58.44861800
H	42.48380500	60.61546500	60.32094700

H	42.82429100	57.94232000	61.66645200
H	41.78266600	61.37673500	62.98868600
H	40.68653700	60.16622400	63.72286300
H	47.33355800	59.60273500	61.19854300

P

C	41.58089300	57.18015200	58.12808900
N	40.86937700	57.54513400	56.92634400
H	41.28221100	57.46268500	55.98818300
H	41.96587200	59.82579300	56.35390300
C	42.85721100	56.36607800	57.95492900
H	40.89485500	56.65772000	58.79985700
C	42.62983300	61.96289600	54.00059000
C	42.75494500	61.28645300	55.37888200
C	44.16964500	60.62464900	55.31737200
C	44.67249900	61.05942900	53.93745700
O	43.95584400	62.22657600	53.60582000
O	44.07742500	59.22052400	55.47103000
H	44.89342600	58.86755100	55.87378400
H	44.80813600	61.04536000	56.09576700
O	41.72308000	60.33989600	55.56160000
C	39.80625800	58.36228700	57.14333100
O	39.45477200	58.66427700	58.28482700
C	39.06425500	58.89519100	55.91976400
N	37.93187700	58.04018600	55.61550400
C	37.98232600	57.02218000	54.70757100
O	37.11065200	56.19673000	54.55263200
H	38.91346300	57.04499000	54.10790900
H	37.14900000	58.02962000	56.25874400
C	38.64521900	60.34052700	56.18658700
C	38.14507900	61.03854400	54.93089200
S	37.62234900	62.75348400	55.23309700
C	39.24124900	63.50215000	55.57416500
H	39.88390000	63.42004300	54.69412500
H	39.06176900	64.55962100	55.77530100
H	39.73551900	63.05569400	56.44058700
H	37.27199200	60.52435100	54.51868900
H	38.92554400	61.03781900	54.16002300
H	37.87750000	60.35290600	56.96711200
H	39.51313100	60.87487300	56.58293400
H	39.73339100	58.85502800	55.05459400
H	42.71302700	62.03210200	56.18211900
C	38.75549600	63.69729700	60.33743600
O	40.09442300	63.27276800	60.46675400

H	40.36111000	62.86183700	59.62273400
O	41.15785500	62.11086000	58.20405700
H	42.05273500	62.45280700	58.44096300
H	41.21852000	61.14988800	58.28278400
O	41.92898000	57.88241400	54.27831800
H	41.72340100	58.82831800	54.38575800
H	42.88239500	57.91408800	54.45065500
H	38.62771400	64.43562900	59.53430800
H	38.06815300	62.86278300	60.14342000
H	38.46063900	64.16718100	61.27889200
H	42.13604500	61.25859000	53.30944100
H	42.07929700	62.90380800	54.01542800
H	45.73959800	61.28522100	53.89716100
H	44.44912300	60.24494500	53.22716800
P	46.08913900	61.29230100	61.68189100
O	45.96535000	62.66699500	61.21600300
O	44.67734100	60.61904600	62.02830600
C	44.48339900	59.20162400	62.14617600
H	44.95734700	58.84784000	63.06891500
H	44.93784500	58.69881800	61.28345000
O	46.82429900	60.24425900	60.68519800
O	47.04522200	61.05418000	62.96812500
N	46.56696700	66.25299300	59.07492400
C	47.56418200	65.40109700	58.65361800
H	48.57886700	65.75919400	58.53863200
N	47.19270700	64.17197900	58.40771900
C	45.83402900	64.15737600	58.67259600
C	44.83111200	63.12859400	58.63365500
N	45.04416700	61.83294100	58.35208000
H	44.23312000	61.23530400	58.27236800
H	45.92918700	61.52365200	57.96527500
N	43.55647900	63.39519700	58.92210900
C	43.26120100	64.62810800	59.33035500
H	42.22486700	64.80713600	59.61130400
N	44.10302800	65.61399600	59.43407700
C	45.39115100	65.40473400	59.09380300
N	49.85437100	59.81989200	58.83663200
H	49.59683200	60.10923400	59.77624200
H	49.68202900	58.82060200	58.75333800
C	51.27190800	60.10203200	58.60802600
H	49.41658500	58.50202200	55.77154300
O	48.13895100	57.73178800	54.34934500
C	46.99617700	56.90577000	54.56677400
C	48.37801900	56.73038300	56.49607100

H	48.69571800	55.68018700	56.46842000
C	46.88512100	56.80983000	56.08987100
H	46.33230700	55.91353500	56.39777100
O	46.24266500	57.92545100	56.65856900
H	46.86708100	58.61231800	56.99987000
O	48.65233800	57.28501300	57.78068300
O	47.31852100	60.07361600	57.78150500
H	48.28905100	60.21278100	57.90729300
H	51.43994400	61.17947600	58.65748400
H	51.94777800	59.61351100	59.32095900
H	51.54210300	59.77231200	57.60264000
H	48.18596900	56.73301900	58.41927700
H	46.56588700	61.26382000	63.78134600
H	46.62301700	67.22395700	59.33435000
C	49.09597200	57.52800200	55.38401500
H	49.96783800	56.98823600	54.99933300
H	47.13394500	55.91173600	54.11593200
H	46.12553400	57.39496100	54.12462200
H	43.60236000	56.93157200	57.38505200
H	43.26460100	56.13602900	58.94133600
H	42.64791800	55.42724100	57.43732600
H	46.99256100	60.02013100	58.69252600
C	42.99466300	58.86585200	62.18644000
C	42.19900100	59.38499300	60.97882300
C	40.88534800	59.99082300	61.54278000
C	41.40202100	60.35002800	62.91898600
O	42.37071300	59.38935800	63.32620800
O	39.77596500	59.11497600	61.63603200
H	39.51095600	58.88836900	60.73129500
H	40.61697100	60.90012700	60.99038500
O	41.93720600	58.27581500	60.13661100
C	41.97904600	58.45794700	58.83131700
O	42.35850500	59.47592500	58.27965500
H	42.75344000	60.15398300	60.43318000
H	42.92765600	57.76855200	62.20703600
H	41.86102900	61.34691700	62.88452400
H	40.59531300	60.35287600	63.65448600
H	47.33354800	59.60276700	61.19850000
